# Asian Admixture in European *Echinococcus multilocularis* Populations: New Data From Poland Comparing EmsB Microsatellite Analyses and Mitochondrial Sequencing

**DOI:** 10.3389/fvets.2020.620722

**Published:** 2021-01-15

**Authors:** Gérald Umhang, Jenny Knapp, Marion Wassermann, Vanessa Bastid, Carine Peytavin de Garam, Franck Boué, Tomasz Cencek, Thomas Romig, Jacek Karamon

**Affiliations:** ^1^Wildlife Surveillance and Eco-Epidemiology Unit, National Reference Laboratory for Echinococcus spp., Rabies and Wildlife Laboratory, ANSES, Malzéville, France; ^2^UMR CNRS 6249 Laboratoire Chrono-Environnement, Université Franche-Comté, Besançon, France; ^3^Department of Parasitology-Mycology, National Reference Centre for Echinococcoses, University Hospital of Besançon, Besançon, France; ^4^Parasitology Unit, University of Hohenheim, Stuttgart, Germany; ^5^Department of Parasitology, National Veterinary Research Institute, Pulawy, Poland

**Keywords:** *Echinococcus multilocularis*, Poland, EmsB microsatellite, mitochondrial sequencing, Asian origin, cross-fertilization

## Abstract

The cestode *Echinococcus multilocularis* is the causative agent of a severe zoonotic disease: alveolar echinococcosis (AE). The parasite is distributed over a vast area in northern Eurasia and North America, but the impact of AE on human health is highly uneven between different regions. One hypothetical reason for this difference in virulence may be the genetic structure of *E. multilocularis* which—based on mitochondrial sequences and EmsB microsatellite profiles—forms four distinct clades. These clades correspond approximately to their continents of origin: Asia, Europe, and North America, with a fourth clade apparently restricted to Mongolia and neighboring regions, even though this clade has not yet been described by EmsB genotyping. However, there are various records of genetic variants from the “wrong” region, e.g., “European” haplotypes in Western Canada, which may be the result of introduction or natural migration of host animals. One such example, prompting this study, is the recent record of an “Asian” mitochondrial haplotype in worms from foxes in Poland. At the time, this could not be confirmed by EmsB microsatellite analysis, a method that has proven to possess greater discriminatory power with the *E. multilocularis* nuclear genome than sequencing of mitochondrial markers. Therefore, worms collected from foxes in Poland were examined both by EmsB analysis and sequencing of the full mitochondrial *cox1* gene in order to allocate the samples to the European or Asian cluster. Based on EmsB analyses of 349 worms from 97 Polish red foxes, 92% of the worms clearly showed “European-type” EmsB profiles, but 27 worms (8%) from seven foxes showed profiles that clustered with samples of Asian origin. According to *cox1* sequences, a total of 18 worms from 8 foxes belonged to the Asian cluster of haplotypes. The two methods did not fully agree: only 13 worms from three foxes belonged to Asian clusters by both EmsB and *cox1*, whereas 18 worms from nine foxes belonged to different clusters, according to each marker. Cross-fertilization between worms of Asian origin and those from the European Polish population may explain these conflicting results. The presence of clearly Asian elements in the Polish *E. multilocularis* population could be the result of introduction of *E. multilocularis* with host animals (e.g., domestic dogs), or the migration of foxes. In the absence of genetic data from eastern European countries, especially those bordering Poland, it cannot be concluded whether this Asian admixture is typical for a larger area toward central/eastern Europe, or the Polish parasite population is the western extreme of a gradient where both European and Asian elements mingle. Further studies are needed on this subject, preferably using both mitochondrial sequencing and EmsB microsatellite analysis.

## Introduction

The cestode *Echinococcus multilocularis* is the causative agent of a severe parasitic disease: alveolar echinococcosis (AE). This zoonotic disease is widely distributed throughout the northern hemisphere. China is considered the main focus of human AE cases, with an estimated 91% of all new cases per year worldwide; in contrast, the European proportion of the global load was estimated at <1%, representing 168 new AE cases annually (1). After the initial infection event, human disease is characterized by a long asymptomatic period (5–15 years), during which parasitic lesions develop in the liver, potentially extending or metastasizing to other organs. The mortality rate is >90% in untreated or inadequately treated cases within 10–15 years after diagnosis (2). Humans become infected through oral ingestion of *E. multilocularis* eggs dispersed into the environment. In the typical parasitic lifecycle, small mammals (often arvicoline rodents) ingest these eggs, leading to the development of hepatic lesions producing protoscoleces. The prey-predator relationship between these rodents and carnivores, in central and eastern Europe mainly red foxes, results in the colonization of the small intestines of carnivores by these protoscoleces, where they evolve to adult worms releasing eggs into the environment via the feces.

The impact of AE on human health is highly uneven between different regions. One hypothetical reason for this difference in virulence may be the genetic structure of *E. multilocularis*. A study based on sequencing of three complete mitochondrial genes *cox1, cob* and *nad2* (3,558 bp) of *E. multilocularis* samples resulted in the identification of four different geographical clades: Europe, Asia and North America, with a fourth clade apparently restricted to Mongolia and neighboring regions (3). The correlation between mitochondrial haplotype groups and geographical areas was recently demonstrated by different studies to be more complex, despite a still limited and inadequate geographical and numerical sampling coverage (4), with various records of genetic variants from the “wrong” region. European mitochondrial haplotypes were identified in Canada (5–8), but also in a captive primate from Russia (Moscow zoo), even though the latter probably did not correspond to autochthonous infection (9). Mongolian and North American mitochondrial haplotypes have been identified in Southern and Northern Siberia, respectively (9). Asian mitochondrial haplotypes were reported from the European part of Russia (9) and northwestern America (Alaska, Saint Lawrence island) (3).

Investigations of the genetic diversity of *E. multilocularis* were simultaneously to mitochondrial sequencing carried out using the EmsB microsatellite marker. This molecular tool has a very high discriminative power due to the quantitative exploitation of the amplification of about 40 copies located on chromosome 5 (10–12). EmsB studies confirmed the same geographical clades as those obtained by mitochondrial sequencing for Europe and Asia, although samples from various circumpolar locations above the Arctic Circle clustered with North American isolates, possibly due to long-distance mobility of Arctic foxes (11, 13, 14). EmsB profiles of the Mongolian clade have currently not been described, most probably due to the absence of EmsB genotyping of samples from this area. As for mitochondrial haplotypes, there are also some samples where the EmsB profiles do not correspond to their geographical origin. One *E. multilocularis* rodent sample from Canada and another from a human patient from Alaska shared the same EmsB profile with a Japanese isolate in the Asian clade (11). The exclusive presence in Svalbard of a single EmsB profile from the Arctic clade is coherent, despite it being geographically a part of Europe (14). Further European studies on the presence of *E. multilocularis* based on EmsB have revealed only profiles clustering together, therefore designated “European” profiles. The expansion of the parasite in Europe was investigated in the historical Alpine focus, with peripheral areas revealing mainland-island transmission ruled by founder events due to migration of red foxes (13). The presence of the parasite across France, but also in Denmark and Sweden, has confirmed this transmission scheme due to the identification of EmsB profiles previously reported from the historical focus in south-central Europe (15, 16). Microsatellite investigations of 301 worms from 87 foxes (one to five worms per fox) originating from all endemic provinces of Poland have resulted in the identification of 29 EmsB profiles and highlighted the influence of neighboring countries in the spatial expansion of the parasite (17). In the same period of the cited study, mitochondrial sequencing (*cox1, cob, nad2*) was carried out on 83 worms isolated from red foxes (one worm per fox) (18), almost all had previously been characterized by EmsB. Seven of these worms, all from the northeastern part of Poland, belonged to a haplotype with very close genetic proximity to haplotypes typical for Asia. However, five of these seven worms were considered to belong to EmsB profiles (Pol01, Pol03, Pol17, and Pol19) typical for Europe, while for technical reasons, no profile was obtained for the other two worms (17).

In many studies using EmsB as a tool to investigate genetic diversity in *E. multilocularis*, attention was focused on the national or regional context, especially in the construction of dendrograms which only include samples from that study. However, due to the nature of the marker and the unweighted pair group method with arithmetic mean (UPGMA) used, the clustering structure of the dendrogram may be influenced by the number and the individual variation of the samples used (19). To obtain information for a larger geographical context, it is therefore necessary to combine larger and spatially distant EmsB data sets.

The identification of mitochondrial haplotypes of the Asian cluster in Poland and the availability of EmsB data from the same worms attributed to the European cluster has prompted this study, focusing on Poland, where the described inconsistencies between mitochondrial sequencing and EmsB results had been observed. Additional sequencing of a mitochondrial target (full *cox1*) was carried out in order to evaluate the current epidemiological situation in Poland regarding a potential Asian admixture in European *E. multilocularis* populations.

## Materials and Methods

### Sample Collection

The 301 worms from 87 Polish foxes previously analyzed for EmsB microsatellite genetic diversity in the studies carried out by Umhang et al. (17) were added to 46 worms from ten foxes genotyped by Knapp et al. (13) to construct a dendrogram that also includes samples of Asian and Arctic origin [i.e., China, Japan, Canada, Alaska, and Svalbard from (11, 14)]. The foxes were each identified by a number and each worm by the number of the fox followed by the number of the individual worm (from one to five), as practiced previously (13, 17). Worms with an Asian mitochondrial haplotype identified previously (18) without available EmsB profiles were re-examined by EmsB genotyping. In addition, EmsB microsatellite data from all other *E. multilocularis* samples previously genotyped [EWET Project, (20)] in Europe (13, 15, 16, 21) and available from the EmsB database (20) were used to visualize potential Asian origins by performing a hierarchical clustering analysis represented in a dendrogram.

### EmsB Microsatellite Analyses and Clustering Dendrogram

EmsB PCR amplification was performed as previously reported (17). Capillary electrophoresis of PCR products was performed on a 3500 genetic analyzer (Life Technologies, Foster City, CA, USA). The size and height of each peak of the electrophoretic profile constituting the EmsB profiles were determined with the use of GeneMapper 4.1. The characterization of EmsB profiles composed of several peaks or alleles from 209 to 241 bp was carried out as previously described (11, 20). The hierarchical clustering analysis was done using the Euclidean distance and the average link clustering method (UPGMA) (22). The uncertainty of clusters was evaluated by multiscale bootstrap resampling (B = 1,000) and given by approximately unbiased *p*-values (AU), according to Shimodaira (23, 24). Clustering analyses were performed using R statistical software (25) and the pvclust library (26). In each dendrogram, EmsB microsatellite data from previously genotyped samples from the Arctic and Asian groups (11, 13) were added. The genetic threshold of 0.08 was used to determine the genotyping status of each sample (11), while two *E. granulosus sensu stricto* (G1) samples were used as the outgroup.

### Mitochondrial Sequencing and Haplotype Analysis

The worm samples with an Asian EmsB profile identified were submitted to full *cox1* sequencing when the haplotype was not previously obtained by Karamon et al. (18). Sequencing of the full mitochondrial *cox1* gene (1,608 bp) was performed from amplicons obtained by PCR, as previously reported (27, 28). Nucleotide sequences of the *cox1* gene obtained were used in addition to those previously reported (3, 9, 18) to construct a TCS haplotype network (29) generated with PopART (http://popart.otago.ac.nz). The previously reported *cox1* haplotypes from Russia (9) and from Europe (Austria, France, Belgium, Slovakia, Germany), Asia (Kazakhstan, China), North America, and Mongolia (China: Inner Mongolia) (3) with their original identification were integrated into this network. Polish *cox1* haplotypes were designated bythe letter referring to the specific *cox1* haplotype [e.g., POL_Efor *cox1* E haplotype from Poland as in (18)].

## Results

**Figure 1:**
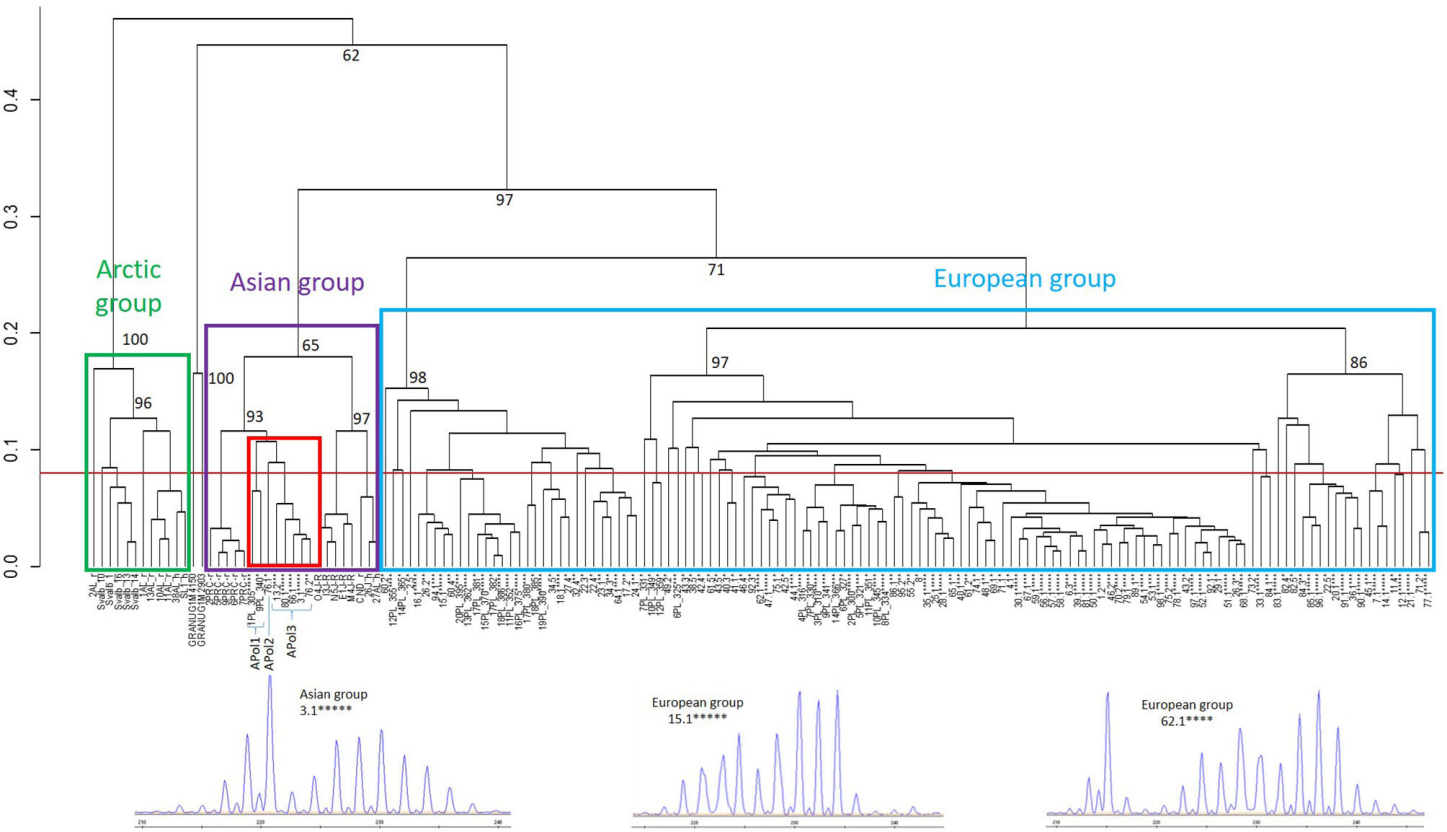
Dendrogram constructed from EmsB data for Polish worm samples from Knapp et al. (13) and Umhang et al. (17). Worms from the same fox with the same profile were pooled to simplify the dendrogram, with the number of asterisks representing the number of worms concerned. Polish samples are indicated by the number of the fox and the representative worm, spaced by PL concerning samples from Knapp et al. (13). The outgroup control is made up of two samples of *Echinococcus granulosus sensu stricto* (G1). Samples from the EWET database were used for Asian (J: Japan, PRC: China, CND: Canada, AL: Alaska) and Arctic groups (AL: Alaska, Svalbard). The Polish samples with Asian profiles APol1 to APol3 are framed in red. The approximately unbiased *p*-values (black numbers on nodes, in percent) were calculated with multiscale bootstrap resampling (B = 1,000). A representative EmsB electrophoregram is provided for one Asian and two European profiles.

The retrospective analyses of 935 EmsB genotyped samples from all European countries except Poland (France, Sweden, Denmark, Estonia, Germany, Switzerland, Czech Republic, Austria, and Slovakia) did not reveal the presence of any Asian EmsB profiles and confirmed the exclusive presence of European profiles (data not shown). A dendrogram was constructed including the 349 worms from 97 Polish red foxes previously genotyped using EmsB (13, 17) and also including the two worms with the Asian cox_E haplotype for *cox1* reported by Karamon et al. (18) (worms 13.1 and 76.1 not available in Umhang et al. (17) (Figure 1). The analysis confirmed that 92% of the Polish worms belonged to the European group, but 27 worms isolated from seven foxes clustered in the three EmsB profiles APol1 to APol3 from the Asian group. These three profiles cluster together and are more distant from the other samples of Asian origin from China and Japan. Profile APol1 is exclusively composed of six worms from two foxes from North Poland (13) (Table 1). Profile APol2 is represented by only one worm (76.1) and is close to profile APol3, which is composed of 20 worms from 6 foxes genotyped in the national Polish study (17), with the exception of worm 13.1 which grouped with other worms from fox #13.

**Table 1:** Results of full *cox1* sequencing and EmsB microsatellite analyses for Polish worm samples of Asian origin identified by at least one of the two molecular methods.

**ID sample (fox_worm)**	**Province of Poland**	**Original study concerned**	**EmsB genotyping**			**Full** ***cox1*** **sequencing**			**Consensus origin**
			**Original profile**	**Study profile**	**Origin**	**Haplotype**	**GENBANK ID**	**Origin**	**EmsB/*cox1***
1PL_305	WM	Knapp et al. (13)	G01	APol1	Asian	POL_B (partial)	NA	European	Asian/European
1PL_306			G01	APol1	Asian	POL_B	MW255891	European	Asian/European
1PL_307			G01	APol1	Asian	NA	NA	NA	Asian/European
1PL_308			G01	APol1	Asian	POL_B	MW255892	European	Asian/European
1PL_309			G01	APol1	Asian	POL_B	MW255893	European	Asian/European
9PL340	WM	Knapp et al. (13)	G01	APol1	Asian	POL_A	MW255894	European	Asian/European
3.1	WM	Umhang et al. (17) and Karamon et al. (18)	P01	Apol3	Asian	POL_E	KY205685	Asian	Asian
3.2		Umhang et al. (17)	P01	Apol3	Asian	POL_E	MW255896	Asian	Asian
3.3			P01	Apol3	Asian	POL_E	MW255897	Asian	Asian
3.4			P01	Apol3	Asian	POL_E (partial)	NA	Asian	Asian
3.5			P01	Apol3	Asian	POL_E	MW255898	Asian	Asian
13.1	LB	Karamon et al. (18) and this study	NA	APol3	Asian	POL_A	KY205677	European	Asian/European
13.2		Umhang et al. (17)	P01	APol3	Asian	POL_A	MW255900	European	Asian/European
13.4			P01	APol3	Asian	POL_A	MW255901	European	Asian/European
66.1	PD	Umhang et al. (17) and Karamon et al. (18)	P01	APol3	Asian	POL_E	KY205685	Asian	Asian
66.2		Umhang et al. (17)	P01	APol3	Asian	POL_E (partial)	NA	Asian	Asian
66.3			P01	APol3	Asian	POL_E	MW255903	Asian	Asian
66.4			P01	APol3	Asian	POL_E	MW255904	Asian	Asian
66.5			P01	APol3	Asian	POL_E	MW255905	Asian	Asian
76.1	KP	Karamon et al. (18) and this study	NA	APol2	Asian	POL_E	KY205685	Asian	Asian
76.2		Umhang et al. (17)	P01	APol3	Asian	POL_E	MW255907	Asian	Asian
76.4			P01	APol3	Asian	POL_E (partial)	NA	Asian	Asian
80.1	MZ	Umhang et al. (17) and Karamon et al. (18)	P01	APol3	Asian	POL_A	KY205677	European	Asian/European
80.2		Umhang et al. (17)	P01	APol3	Asian	POL_A	MW255909	European	Asian/European
80.3			P01	APol3	Asian	POL_A	MW255910	European	Asian/European
80.4			P01	APol3	Asian	POL_A	MW255911	European	Asian/European
80.5			P01	APol3	Asian	NA	NA	NA	NA
40.1	WM	Umhang et al. (17) and Karamon et al. (18)	P19	EPol26	European	POL_E	KY205685	Asian	European/Asian
44.1	WM	Umhang et al. (17) and Karamon et al. (18)	P17	EPol22	European	POL_E	KY205685	Asian	European/Asian
77.1	MZ	Umhang et al. (17) and Karamon et al. (18)	P03	EPol34	European	POL_E	KY205685	Asian	European/Asian
78.1	MZ	Umhang et al. (17) and Karamon et al. (18)	P19	EPol26	European	POL_E	KY205685	Asian	European/Asian
45.1	WM	Umhang et al. (17) and Karamon et al. (18)	P24	EPol31	European	POL_E	KY205685	Asian	European/Asian

**Figure 2:**
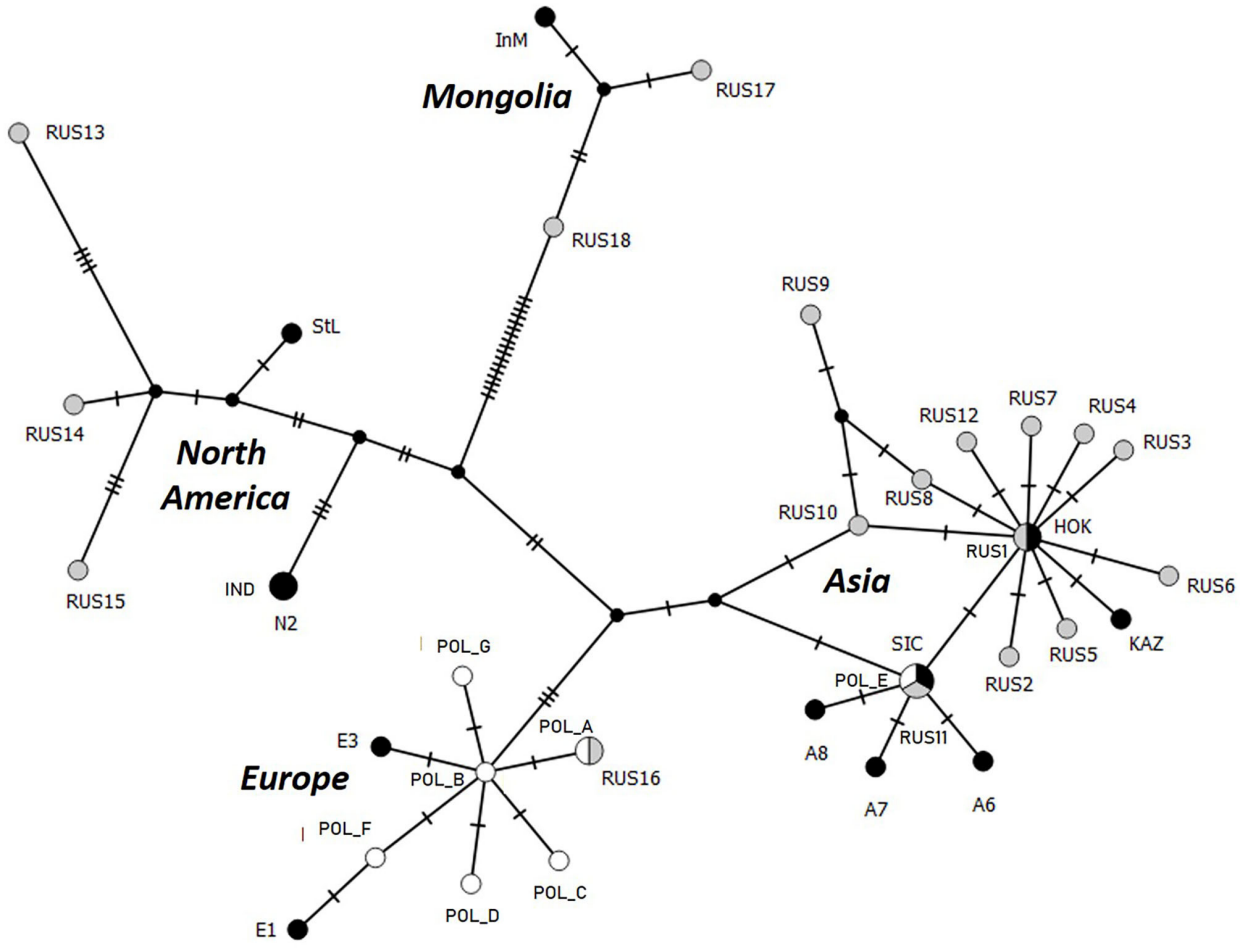
Parsimonious network of *cox1* haplotypes of *E. multilocularis* samples from Poland (white) completed by others previously reported by Konyaev et al. (9) (gray) and Nakao et al. (3) (black), with their original identification of haplotypes. E, Europe; POL, Poland; RUS, Russia; InM, Chinese Inner Mongolia; StL, Alaska (St-Lawrence Island); SIC, China (Sichuan); HOK, Japan (Hokkaido); KAZ, Kazakhstan.

In addition to the ten nucleotide sequences of the full *cox1* gene already available (18), 19 others (including four partial sequences) were obtained from worms with an Asian EmsB profile (Table 1). The three *cox1* haplotypes A, B, and E previously reported by Karamon et al. (18) were identified in the 27 worms from the seven foxes, with the same haplotype for worms from the same fox. Haplotypes A and B of the European group differ in only one mutation and were identified in worms from foxes #1PL, #9PL, #13, and #80 (Figure 2). Haplotype E corresponds to a previously reported haplotype from Sichuan (China) and Altai (Russia) in the Asian group, and was identified in worms from foxes #3, #66, and #76. Karamon et al. (18) reported this haplotype previously from one worm each from five different foxes (40.1, 44.1, 77.1, 78.1, and 45.1), all of them showing European EmsB profiles, but also from one worm each from four different foxes (3.1, 13.1, 66.1, 76.1) showing Asian EmsB profiles (APol2 and Apol3), like the other worms from these foxes.

In total, worms from 12 foxes harbor an Asian mitochondrial haplotype and/or an Asian EmsB profile (Table 1, Supplementary Table 1). Thirteen worms isolated from three foxes (#3, #66, #76) belonged to Asian clusters by both EmsB and *cox1*. An Asian EmsB profile but a European mitochondrial haplotype was found in 13 worms from four foxes (#1PL, #9PL, #13, and #80), whereas an Asian mitochondrial haplotype and a European EmsB profile was obtained from the remaining five foxes. The geographic location of the foxes harboring worms from the Asian cluster (*cox1* haplotype and/or EmsB) concerned the provinces of Lubuskie (LB), Warmińsko-Mazurskie (WM), Kujawsko-Pomorskie (KP), Podlaskie (PD), and Mazowieckie (MZ).

## Discussion

Genetic variants of *E. multilocularis* from the “wrong” regions had previously been described from North America and Asia, but this had never before concerned samples from non-Arctic Europe until the identification of a mitochondrial haplotype of Asian origin in Polish worms (18). Previous genetic analyses performed by mitochondrial sequencing had consistently identified European haplotypes in European samples (3, 21). In the same way, all the EmsB profiles previously reported from Europe were attributed to the European cluster (13, 15, 16, 20, 21). Here for the first time, both methods (representing the nuclear and the mitochondrial genomes) were used consistently with a larger set of samples. The results reveal the widespread and rather frequent presence in Poland of variants that belong to mitochondrial and/or EmsB clusters typical for Asia, and not known from anywhere else in Europe. Erroneously, profiles of certain samples had previously been allocated to the European cluster of EmsB profiles (13, 17), which could be shown to be an artifact due to the absence of samples from the Asian group in the dendrograms in that study. Attribution to one of the different clades (Europe, Asia, Arctic) using EmsB analyses requires systematic visual analysis of the electrophoretic profile and a hierarchical clustering analysis resulting in a dendrogram that includes samples from each of the different clades: Europe, Asia, and Arctic (including North America). Mitochondrial sequencing, unexpectedly, did not in all cases correspond to the EmsB status regarding the European or Asian clades. Only 13 worms from three foxes belonged to Asian clusters by both EmsB and *cox1*, whereas 18 worms from nine foxes belonged to different clusters according to each marker. However, the large majority (92%) of worms from Poland belonged to European clusters by both methods.

The two methods target mitochondrial or nuclear genomes corresponding to coding and non-coding regions, respectively. In contrast to the mitochondrial genome, the nuclear genome is subjected to recombination and is inherited by both male and female. Recent studies have confirmed that both cross-fertilization and self-fertilization occur within species of *Echinococcus*, including *E. multilocularis* (11, 30–32). Cross-fertilization between worms from two different strains may occur in the intestines, leading to production of eggs with the mitochondrial genome of female origin, but with a nuclear genome integrating genetic material of male origin (“male introgression”). Worms with discrepancies between mitochondrial sequencing and EmsB microsatellite analyses are assumed to result from cross-fertilization between worms of typically “European” and typically “Asian” genomes.

The presence of clusters of genetic variants (both mitochondrial and nuclear) that correspond to continental origins indicates prolonged evolution in these geographical areas, without significant genetic exchange. Our study from Poland is the first large-scale investigation where a zone of apparent overlap or co-existence of such variants was identified. Our data do not allow for an unequivocal explanation of this observation, i.e., whether the presence of different variants was caused by recent introduction (e.g., via traveling domestic dogs, or via migration of wild foxes), or whether this situation represents an ancient polymorphism that has been present in the area for a longer period. The presence of only three EmsB profiles and one mitochondrial haplotype of the Asian cluster argues for (a) sporadic introduction event(s), although our observation of a “mosaic” distribution of mitochondrial and nuclear variants appears to indicate prolonged presence of these variants in Poland with sufficient time for recombination. However, it cannot be ruled out that the Polish worms are the westernmost representatives of Asian variants, and there may be a gradient of progressively decreasing “Asian” components in the genome of *E. multilocularis* populations from East to West. To decide on this, additional genetic analyses in Western Asia and Eastern Europe will be needed. To date, only few data are available from this region: European haplotypes and EmsB profiles were obtained from worms isolated in raccoon dogs from Estonia (21), but no genetic data are available for neighboring countries such as Latvia, Belarus and Ukraine. Concerning Russia, the only *E. multilocularis* sample reported that belongs to the European cluster (according to full *cox1* sequencing) was from a captive primate (*Galago senegalensis*) from the Moscow zoo, where there was a strong suspicion of an infection source from Baltic countries through imported mulch spread as ground cover in the enclosure (9). All the other *E. multilocularis* samples from the European or Asian parts of the Russian Federation belonged to Asian, North American or Mongolian genetic clusters.

It will be interesting to study the geographical limits of this Asian admixture in European *E. multilocularis* populations outside of Poland. As shown in this study, the simultaneous use of both mitochondrial sequencing and EmsB analysis is relevant in order to increase sensitivity and to detect introgression events. As we mentioned, future studies will be particularly relevant in the eastern part of Europe, but sample sizes using the described approach are not large, even in well-known endemic areas of central Europe. Given the fact that Asian genetic components in Poland were found not to be limited to the Northeast of the country but extend as far as Lubuskie (LB) province on the border with Germany, it would not be surprising to detect this kind of admixture even further to the West.

It has been speculated that the presence of certain genetic variants of *E. multilocularis* may have an impact on public health via differences in infectivity or pathogenicity to humans (33). This hypothesis has for instance served as a hypothetical explanation for the low number of human AE cases in North America, despite widespread presence of the parasite in animal hosts. However, no conclusions have been reached on this, and our detection of widespread recombinations between nuclear and mitochondrial markers calls for an examination of human samples using both approaches, as pathogenicity factors will most likely be situated in the nuclear genome rather than the mitochondria, which are at present far more frequently used for genetic characterization of isolates and allocation to clusters. This is highly relevant for the area under study, as, like elsewhere in Europe, an increasing prevalence in red foxes was observed followed by an increase of AE human cases associated with high morbidity and mortality, resulting in a public health situation that is of concern (34, 35).

## Data Availability Statement

The datasets presented in this study can be found in online repositories. The names of the repository/repositories and accession number(s) can be found in the article/Supplementary Material.

## Author Contributions

GU, JKa, JKn, and TR: conceptualization. GU, JKa, and JKn: methodology. VB, CP, MW, GU, and JKa: investigation. JKa, TC, JKn, TR, MW, and GU: ressources. GU: writing original draft preparation and project administration. JKn, JKa, TR, MW, and FB: writing-review and editing. All authors contributed to the article and approved the submitted version.

## Conflict of Interest

The authors declare that the research was conducted in the absence of any commercial or financial relationships that could be construed as a potential conflict of interest. The handling editor declared a past co-authorship with the authors TR and MW.
